# 5-Bromo-2-methyl-3-phenyl­sulfonyl-1-benzofuran

**DOI:** 10.1107/S1600536808008489

**Published:** 2008-04-02

**Authors:** Hong Dae Choi, Pil Ja Seo, Byeng Wha Son, Uk Lee

**Affiliations:** a Department of Chemistry, Dongeui University, San 24 Kaya-dong, Busanjin-gu, Busan 614-714, Republic of Korea; b Department of Chemistry, Pukyong National University, 599-1 Daeyeon 3-dong, Nam-gu, Busan 608-737, Republic of Korea

## Abstract

The title compound, C_15_H_11_BrO_3_S, was prepared by the oxidation of 5-bromo-2-methyl-3-phenyl­sulfanyl-1-benzofuran with 3-chloro­peroxy­benzoic acid. The phenyl ring makes a dihedral angle of 78.99 (8)° with the plane of the benzofuran fragment. The crystal structure is stabilized by C—H⋯π inter­actions between a benzene H atom of the benzofuran unit and the phenyl ring of the phenyl­sulfonyl substituent from a neighbouring mol­ecule. In addition, the crystal structure exhibits intra- and inter­molecular C—H⋯O inter­actions.

## Related literature

For the crystal structures of similar 5-bromo-2-methyl-1-benzofuran derivatives, see: Choi *et al.* (2007 ▶); Seo *et al.* (2007 ▶).
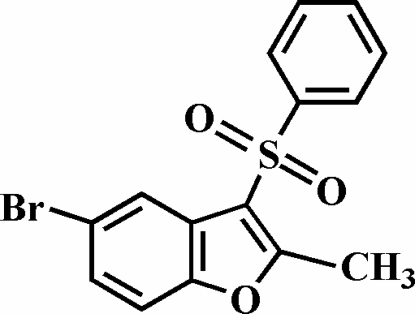


## Experimental

### 

#### Crystal data

C_15_H_11_BrO_3_S
*M* *_r_* = 351.21
Monoclinic, 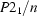

*a* = 7.337 (1) Å
*b* = 11.345 (1) Å
*c* = 16.602 (2) Å
β = 94.582 (3)°
*V* = 1377.5 (3) Å^3^
*Z* = 4
Mo *K*α radiation
μ = 3.14 mm^−1^
*T* = 173 (2) K
0.30 × 0.20 × 0.20 mm

#### Data collection

Bruker SMART CCD diffractometer
Absorption correction: multi-scan (*SADABS*; Sheldrick, 1999 ▶) *T*_min_ = 0.463, *T*_max_ = 0.542
8005 measured reflections
3009 independent reflections
2349 reflections with *I* > 2σ(*I*)
*R*_int_ = 0.033

#### Refinement

*R*[*F* ^2^ > 2σ(*F* ^2^)] = 0.033
*wR*(*F* ^2^) = 0.086
*S* = 1.03
3009 reflections
182 parameters
H-atom parameters constrained
Δρ_max_ = 0.55 e Å^−3^
Δρ_min_ = −0.44 e Å^−3^

### 

Data collection: *SMART* (Bruker, 2001 ▶); cell refinement: *SAINT* (Bruker, 2001 ▶); data reduction: *SAINT*; program(s) used to solve structure: *SHELXS97* (Sheldrick, 2008 ▶); program(s) used to refine structure: *SHELXL97* (Sheldrick, 2008 ▶); molecular graphics: *ORTEP-3* (Farrugia, 1997 ▶) and *DIAMOND* (Brandenburg, 1998 ▶); software used to prepare material for publication: *SHELXL97*.

## Supplementary Material

Crystal structure: contains datablocks global, I. DOI: 10.1107/S1600536808008489/cf2191sup1.cif

Structure factors: contains datablocks I. DOI: 10.1107/S1600536808008489/cf2191Isup2.hkl

Additional supplementary materials:  crystallographic information; 3D view; checkCIF report

## Figures and Tables

**Table 1:** Hydrogen-bond geometry (Å, °)

*D*—H⋯*A*	*D*—H	H⋯*A*	*D*⋯*A*	*D*—H⋯*A*
C6—H6⋯*Cg*^i^	0.95	2.74	3.561 (3)	145
C10—H10⋯O2^ii^	0.95	2.54	3.285 (3)	135
C14—H14⋯O3^iii^	0.95	2.45	3.249 (3)	141
C15—H15*C*⋯O3	0.98	2.40	3.131 (4)	131

Symmetry codes: (i) 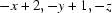; (ii) 
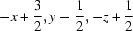
; (iii) 
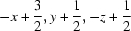
. *Cg* is the centroid of the phenyl ring C9–C14.

**Fig. 1.**
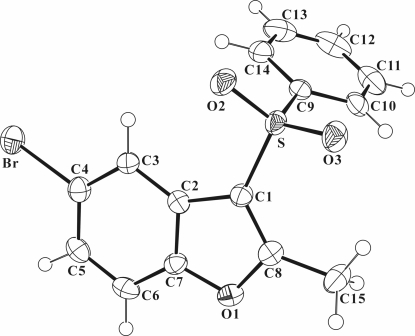
The molecular structure of the title compound, showing displacement ellipsoides drawn at the 50% probability level.

**Fig. 2.**
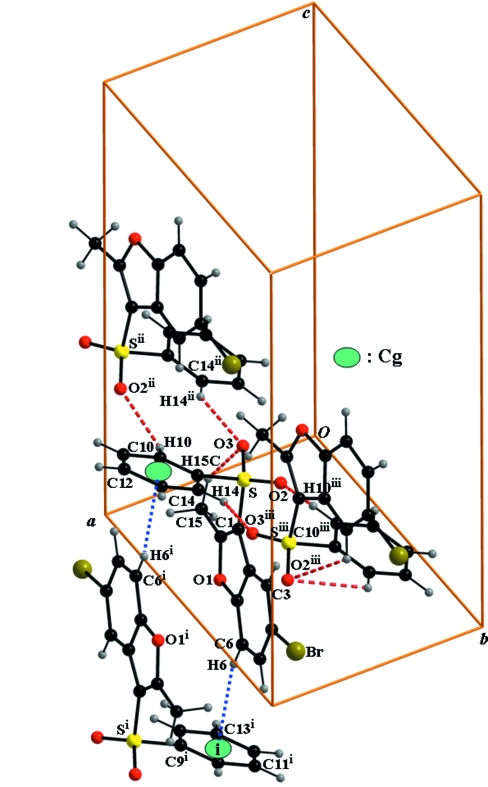
C—H···π and C—H···O interactions (dotted lines) in the title compound. Cg denotes the ring centroid. [Symmetry code: (i) -x + 2, -y + 1, -z; (ii) -x + 3/2, y - 1/2, -z + 1/2; (iii) -x + 3/2, y + 1/2, -z + 1/2.]

## supplementary crystallographic information

### Comment

This work is related to earlier communications on the synthesis and structure of 5-bromo-2-methyl-1-benzofuran analogues, *viz*. 5-bromo-2-methyl-3-methylsulfinyl-1-benzofuran (Choi *et al.*, 2007) and 5-bromo-2-methyl-3-phenylsulfinyl-1-benzofuran (Seo *et al.*, 2007). Here we report the crystal structure of the title compound, 5-bromo-2-methyl-3-phenylsulfonyl-1-benzofuran (Fig. 1).

The benzofuran unit is essentially planar, with a mean deviation of 0.007 Å from the least-squares plane defined by the nine constituent atoms. The phenyl ring (C9–C14) makes a dihedral angle of 78.99 (8)° with the plane of the benzofuran fragment. The crystal packing (Fig. 2) is stabilized by intermolecular C—H···π interactions between a benzene H atom of the benzofuran unit and the phenyl ring of the phenylsulfonyl substituent from an adjacent molecule, with a C6—H6···*Cg*^i^ separation of 2.74 Å (Fig. 2; *Cg* is the centroid of the C9–C14 phenyl ring, symmetry code as in Fig. 2). Additionally, intra- and intermolecular C—H···O interactions in the structure are observed (Fig. 2).

### Experimental

3-Chloroperoxybenzoic acid (77%, 336 mg, 1.5 mmol) was added in small portions to a stirred solution of 5-bromo-2-methyl-3-phenylsulfonyl-1-benzofuran (223 mg, 0.7 mmol) in dichloromethane (30 ml) at 273 K. After being stirred for 4 h at room temperature, the mixture was washed with saturated sodium bicarbonate solution and the organic layer was separated, dried over magnesium sulfate, filtered and concentrated in vacuum. The residue was purified by column chromatography (hexane-ethyl acetate, 2:1 *v*/*v*) to afford the title compound as a colorless solid [yield 78%, m.p. 464–465 K; Rf = 0.59 (hexane-ethyl acetate, 2:1 *v*/*v*)]. Single crystals suitable for X-ray diffraction were prepared by evaporation of a solution of the title compound in chloroform at room temperature. Spectroscopic analysis: ^1^H NMR (CDCl_3_, 400 MHz) δ 2.80 (s, 3H), 7.29 (d, J = 8.44 Hz, 1H), 7.41 (dd, J = 8.44 Hz and J = 1.84 Hz, 1H), 7.51–7.56 (m, 2H), 7.58–7.61 (m, 1H), 7.98–8.02 (m, 2H), 8.05 (d, J = 2.20 Hz, 1H); EI—MS 352 [*M*+2], 350 [*M*^+^].

### Refinement

All H atoms were geometrically positioned and refined using a riding model, with C—H = 0.95 Å for aromatic H atoms and 0.98 Å for methyl H atoms, respectively, and with *U*_iso_(H) = 1.2U_eq_(C) for aromatic and *U*_iso_(H) = 1.5U_eq_(C) for methyl H atoms.

### Crystal data

**Table:**

C_15_H_11_BrO_3_S	*F*_000_ = 704
*M**_r_* = 351.21	*D*_x_ = 1.693 Mg m^−^^3^
Monoclinic, *P*2_1_/*n*	Mo *K*α radiation λ = 0.71073 Å
Hall symbol: -P_2yn	Cell parameters from 3065 reflections
*a* = 7.337 (1) Å	θ = 2.5–27.7º
*b* = 11.345 (1) Å	µ = 3.14 mm^−^^1^
*c* = 16.602 (2) Å	*T* = 173 (2) K
β = 94.582 (3)º	Block, colorless
*V* = 1377.5 (3) Å^3^	0.30 × 0.20 × 0.20 mm
*Z* = 4	

### Data collection

**Table:**

Bruker SMART CCD diffractometer	3009 independent reflections
Radiation source: fine-focus sealed tube	2349 reflections with *I* > 2σ(*I*)
Monochromator: graphite	*R*_int_ = 0.033
Detector resolution: 10.0 pixels mm^-1^	θ_max_ = 27.0º
*T* = 173(2) K	θ_min_ = 2.5º
φ and ω scans	*h* = −9→9
Absorption correction: multi-scan(SADABS; Sheldrick, 1999)	*k* = −14→13
*T*_min_ = 0.463, *T*_max_ = 0.542	*l* = −14→21
8005 measured reflections	

### Refinement

**Table:**

Refinement on *F*^2^	Secondary atom site location: difference Fourier map
Least-squares matrix: full	Hydrogen site location: difference Fourier map
*R*[*F*^2^ > 2σ(*F*^2^)] = 0.033	H-atom parameters constrained
*wR*(*F*^2^) = 0.086	*w* = 1/[σ^2^(*F*_o_^2^) + (0.0401*P*)^2^ + 0.6448*P*] where *P* = (*F*_o_^2^ + 2*F*_c_^2^)/3
*S* = 1.03	(Δ/σ)_max_ < 0.001
3009 reflections	Δρ_max_ = 0.55 e Å^−^^3^
182 parameters	Δρ_min_ = −0.44 e Å^−^^3^
Primary atom site location: structure-invariant direct methods	Extinction correction: none

### Special details

**Table:**

Geometry. All e.s.d.'s (except the e.s.d. in the dihedral angle between two l.s. planes) are estimated using the full covariance matrix. The cell e.s.d.'s are taken into account individually in the estimation of e.s.d.'s in distances, angles and torsion angles; correlations between e.s.d.'s in cell parameters are only used when they are defined by crystal symmetry. An approximate (isotropic) treatment of cell e.s.d.'s is used for estimating e.s.d.'s involving l.s. planes.
Refinement. Refinement of *F*^2^ against ALL reflections. The weighted *R*-factor *wR* and goodness of fit *S* are based on *F*^2^, conventional *R*-factors *R* are based on *F*, with *F* set to zero for negative *F*^2^. The threshold expression of *F*^2^ > σ(*F*^2^) is used only for calculating *R*-factors(gt) *etc*. and is not relevant to the choice of reflections for refinement. *R*-factors based on *F*^2^ are statistically about twice as large as those based on *F*, and *R*- factors based on ALL data will be even larger.

### Fractional atomic coordinates and isotropic or equivalent isotropic displacement parameters (Å^2^)

**Table:**

	*x*	*y*	*z*	*U*_iso_*/*U*_eq_	
Br	0.79772 (5)	0.88457 (2)	0.038216 (19)	0.03963 (12)	
S	0.65781 (9)	0.40138 (5)	0.19581 (4)	0.02489 (16)	
O1	0.7486 (3)	0.36914 (16)	−0.03375 (11)	0.0296 (4)	
O2	0.5677 (3)	0.50746 (16)	0.21839 (12)	0.0327 (5)	
O3	0.5740 (3)	0.28903 (15)	0.20838 (12)	0.0344 (5)	
C1	0.6960 (4)	0.4145 (2)	0.09406 (16)	0.0242 (5)	
C2	0.7309 (3)	0.5236 (2)	0.05267 (15)	0.0227 (5)	
C3	0.7410 (4)	0.6429 (2)	0.07353 (16)	0.0255 (5)	
H3	0.7209	0.6693	0.1264	0.031*	
C4	0.7819 (4)	0.7208 (2)	0.01307 (17)	0.0287 (6)	
C5	0.8107 (4)	0.6854 (2)	−0.06521 (16)	0.0305 (6)	
H5	0.8372	0.7425	−0.1045	0.037*	
C6	0.8010 (4)	0.5675 (3)	−0.08587 (16)	0.0308 (6)	
H6	0.8198	0.5412	−0.1389	0.037*	
C7	0.7626 (3)	0.4899 (2)	−0.02563 (16)	0.0245 (5)	
C8	0.7097 (4)	0.3254 (2)	0.03977 (16)	0.0271 (6)	
C9	0.8786 (4)	0.3998 (2)	0.24546 (16)	0.0259 (6)	
C10	0.9716 (4)	0.2932 (2)	0.25599 (16)	0.0321 (6)	
H10	0.9160	0.2218	0.2368	0.039*	
C11	1.1457 (5)	0.2921 (3)	0.29469 (19)	0.0446 (8)	
H11	1.2105	0.2200	0.3024	0.054*	
C12	1.2249 (5)	0.3974 (3)	0.32221 (19)	0.0506 (9)	
H12	1.3445	0.3969	0.3488	0.061*	
C13	1.1314 (5)	0.5031 (3)	0.31132 (19)	0.0467 (8)	
H13	1.1870	0.5745	0.3304	0.056*	
C14	0.9580 (4)	0.5048 (2)	0.27289 (17)	0.0333 (6)	
H14	0.8935	0.5771	0.2652	0.040*	
C15	0.6972 (4)	0.1947 (2)	0.04402 (19)	0.0374 (7)	
H15A	0.8193	0.1605	0.0419	0.056*	
H15B	0.6169	0.1657	−0.0017	0.056*	
H15C	0.6471	0.1719	0.0947	0.056*	

### Atomic displacement parameters (Å^2^)

**Table:**

	*U*^11^	*U*^22^	*U*^33^	*U*^12^	*U*^13^	*U*^23^
Br	0.0526 (2)	0.02501 (16)	0.0412 (2)	−0.00489 (13)	0.00320 (14)	0.00437 (12)
S	0.0301 (4)	0.0189 (3)	0.0269 (3)	−0.0004 (2)	0.0096 (3)	0.0010 (2)
O1	0.0328 (11)	0.0301 (10)	0.0263 (10)	0.0005 (8)	0.0042 (8)	−0.0057 (8)
O2	0.0393 (12)	0.0266 (9)	0.0341 (11)	0.0064 (8)	0.0149 (9)	−0.0020 (8)
O3	0.0392 (12)	0.0259 (10)	0.0398 (12)	−0.0066 (8)	0.0140 (9)	0.0038 (8)
C1	0.0256 (14)	0.0230 (12)	0.0246 (13)	0.0000 (10)	0.0059 (11)	−0.0002 (10)
C2	0.0191 (13)	0.0259 (12)	0.0235 (13)	0.0019 (10)	0.0038 (10)	0.0020 (10)
C3	0.0302 (14)	0.0242 (12)	0.0224 (13)	0.0021 (10)	0.0046 (11)	−0.0006 (10)
C4	0.0273 (15)	0.0262 (13)	0.0325 (15)	−0.0024 (11)	0.0019 (12)	0.0024 (11)
C5	0.0288 (15)	0.0371 (15)	0.0258 (14)	0.0000 (12)	0.0039 (11)	0.0081 (12)
C6	0.0296 (15)	0.0427 (15)	0.0202 (14)	0.0016 (12)	0.0033 (11)	−0.0001 (12)
C7	0.0224 (13)	0.0252 (12)	0.0258 (14)	0.0019 (10)	0.0007 (11)	−0.0038 (10)
C8	0.0243 (14)	0.0275 (13)	0.0298 (15)	−0.0014 (11)	0.0032 (11)	−0.0022 (11)
C9	0.0337 (15)	0.0256 (13)	0.0196 (13)	−0.0007 (10)	0.0096 (11)	0.0012 (10)
C10	0.0420 (18)	0.0317 (14)	0.0239 (14)	0.0033 (12)	0.0101 (12)	0.0077 (11)
C11	0.043 (2)	0.059 (2)	0.0330 (17)	0.0144 (16)	0.0095 (14)	0.0149 (15)
C12	0.0376 (19)	0.087 (3)	0.0264 (17)	−0.0062 (18)	0.0010 (14)	0.0063 (17)
C13	0.053 (2)	0.059 (2)	0.0276 (16)	−0.0175 (17)	0.0033 (14)	−0.0068 (15)
C14	0.0444 (18)	0.0305 (14)	0.0258 (14)	−0.0048 (12)	0.0087 (13)	−0.0023 (11)
C15	0.0437 (19)	0.0249 (14)	0.0443 (18)	−0.0021 (12)	0.0074 (14)	−0.0072 (12)

### Geometric parameters (Å, °)

**Table:**

Br—C4	1.906 (3)	C6—H6	0.950
S—O2	1.4372 (18)	C8—C15	1.487 (4)
S—O3	1.4377 (18)	C9—C14	1.387 (4)
S—C1	1.740 (3)	C9—C10	1.393 (4)
S—C9	1.758 (3)	C10—C11	1.383 (4)
O1—C8	1.369 (3)	C10—H10	0.950
O1—C7	1.380 (3)	C11—C12	1.389 (5)
C1—C8	1.363 (4)	C11—H11	0.950
C1—C2	1.448 (3)	C12—C13	1.387 (5)
C2—C7	1.392 (3)	C12—H12	0.950
C2—C3	1.398 (3)	C13—C14	1.377 (4)
C3—C4	1.388 (4)	C13—H13	0.950
C3—H3	0.950	C14—H14	0.950
C4—C5	1.392 (4)	C15—H15A	0.980
C5—C6	1.381 (4)	C15—H15B	0.980
C5—H5	0.950	C15—H15C	0.980
C6—C7	1.378 (4)		
			
O2—S—O3	119.57 (12)	C1—C8—O1	110.7 (2)
O2—S—C1	107.14 (12)	C1—C8—C15	134.4 (3)
O3—S—C1	108.70 (12)	O1—C8—C15	114.9 (2)
O2—S—C9	108.19 (12)	C14—C9—C10	121.1 (3)
O3—S—C9	108.15 (12)	C14—C9—S	119.5 (2)
C1—S—C9	104.02 (12)	C10—C9—S	119.5 (2)
C8—O1—C7	106.97 (19)	C11—C10—C9	119.4 (3)
C8—C1—C2	107.1 (2)	C11—C10—H10	120.3
C8—C1—S	127.2 (2)	C9—C10—H10	120.3
C2—C1—S	125.57 (19)	C10—C11—C12	119.5 (3)
C7—C2—C3	119.2 (2)	C10—C11—H11	120.3
C7—C2—C1	104.9 (2)	C12—C11—H11	120.3
C3—C2—C1	135.9 (2)	C13—C12—C11	120.7 (3)
C4—C3—C2	116.6 (2)	C13—C12—H12	119.6
C4—C3—H3	121.7	C11—C12—H12	119.6
C2—C3—H3	121.7	C14—C13—C12	120.1 (3)
C3—C4—C5	123.3 (2)	C14—C13—H13	119.9
C3—C4—Br	118.4 (2)	C12—C13—H13	119.9
C5—C4—Br	118.32 (19)	C13—C14—C9	119.2 (3)
C6—C5—C4	120.1 (2)	C13—C14—H14	120.4
C6—C5—H5	119.9	C9—C14—H14	120.4
C4—C5—H5	119.9	C8—C15—H15A	109.5
C7—C6—C5	116.6 (2)	C8—C15—H15B	109.5
C7—C6—H6	121.7	H15A—C15—H15B	109.5
C5—C6—H6	121.7	C8—C15—H15C	109.5
C6—C7—O1	125.5 (2)	H15A—C15—H15C	109.5
C6—C7—C2	124.2 (2)	H15B—C15—H15C	109.5
O1—C7—C2	110.3 (2)		
			
O2—S—C1—C8	−152.5 (2)	C3—C2—C7—O1	179.2 (2)
O3—S—C1—C8	−22.0 (3)	C1—C2—C7—O1	0.1 (3)
C9—S—C1—C8	93.1 (3)	C2—C1—C8—O1	−0.8 (3)
O2—S—C1—C2	31.8 (3)	S—C1—C8—O1	−177.14 (19)
O3—S—C1—C2	162.3 (2)	C2—C1—C8—C15	177.1 (3)
C9—S—C1—C2	−82.7 (2)	S—C1—C8—C15	0.8 (5)
C8—C1—C2—C7	0.4 (3)	C7—O1—C8—C1	0.8 (3)
S—C1—C2—C7	176.8 (2)	C7—O1—C8—C15	−177.5 (2)
C8—C1—C2—C3	−178.5 (3)	O2—S—C9—C14	−21.6 (2)
S—C1—C2—C3	−2.0 (5)	O3—S—C9—C14	−152.5 (2)
C7—C2—C3—C4	0.3 (4)	C1—S—C9—C14	92.0 (2)
C1—C2—C3—C4	179.1 (3)	O2—S—C9—C10	158.9 (2)
C2—C3—C4—C5	0.5 (4)	O3—S—C9—C10	28.0 (2)
C2—C3—C4—Br	−179.92 (19)	C1—S—C9—C10	−87.4 (2)
C3—C4—C5—C6	−0.6 (4)	C14—C9—C10—C11	0.2 (4)
Br—C4—C5—C6	179.8 (2)	S—C9—C10—C11	179.7 (2)
C4—C5—C6—C7	−0.2 (4)	C9—C10—C11—C12	−0.2 (4)
C5—C6—C7—O1	−179.4 (2)	C10—C11—C12—C13	0.1 (5)
C5—C6—C7—C2	1.1 (4)	C11—C12—C13—C14	0.0 (5)
C8—O1—C7—C6	179.8 (3)	C12—C13—C14—C9	0.1 (4)
C8—O1—C7—C2	−0.6 (3)	C10—C9—C14—C13	−0.2 (4)
C3—C2—C7—C6	−1.2 (4)	S—C9—C14—C13	−179.6 (2)
C1—C2—C7—C6	179.7 (3)		

### Hydrogen-bond geometry (Å, °)

**Table:**

*D*—H···*A*	*D*—H	H···*A*	*D*···*A*	*D*—H···*A*
C6—H6···Cg^i^	0.95	2.74	3.561 (3)	145
C10—H10···O2^ii^	0.95	2.54	3.285 (3)	135
C14—H14···O3^iii^	0.95	2.45	3.249 (3)	141
C15—H15C···O3	0.98	2.40	3.131 (4)	131

Symmetry codes: (i) −*x*+2, −*y*+1, −*z*; (ii) −*x*+3/2, *y*−1/2, −*z*+1/2; (iii) −*x*+3/2, *y*+1/2, −*z*+1/2.
